# Colorectal cancer chemoprevention: is aspirin still in the game?

**DOI:** 10.1080/15384047.2022.2104561

**Published:** 2022-07-29

**Authors:** Adrien Grancher, Pierre Michel, Frederic Di Fiore, David Sefrioui

**Affiliations:** a Normandy Centre for Genomic and Personalized Medicine and Department of Hepatogastroenterology, Normandie Univ, Iron Group, Rouen University Hospital, Rouen, France; b Normandy Centre for Genomic and Personalized Medicine, Department of Hepatogastroenterology and Department of Medical Oncology, Henri Becquerel Centre, Normandie Univ, IRON group, Rouen University Hospital, Rouen, France

**Keywords:** Aspirin, colorectal cancer, *PIK3CA* mutation

## Abstract

Screening strategies have demonstrated their potential for decreasing the incidence and mortality of cancers, particularly that of colorectal cancer (CRC). Another strategy that has been developed to reduce CRC occurrence is the use of chemoprevention agents. Among them, aspirin is the most promising. Aspirin acts in colorectal tumourigenesis through several mechanisms, either directly in tumor cells or in their microenvironment, such as through its anti-inflammatory activity or its effect on the modulation of platelet function. Many retrospective studies, as well as follow-up of large cohorts from trials with primary cardiovascular end points, have shown that long-term treatment with daily low-dose aspirin decreases the incidence of adenomas and colorectal cancers. Therefore, aspirin is currently recommended by the United States Preventive Services Task Force (USPSTF) for primary prevention of CRC in all patients aged 50 to 59 with a 10-y risk of cardiovascular events greater than 10%. Furthermore, several studies have also reported that long-term aspirin treatment taking after CRC resection decreases recurrence risk and increases overall survival, especially in patients with *PIK3CA*-mutated tumors. This review summarizes current knowledge on the pathophysiological mechanisms of aspirin chemoprevention, discusses the primary clinical results on CRC prevention and highlights the potential biomarkers identified to predict aspirin efficacy.

## Introduction

Colorectal cancer (CRC) is a worldwide public health problem. Every year, more than 1.8 million CRCs are diagnosed, and 800,000 persons die from this disease.^1^ Screening strategies for CRC have also made it possible to decrease both the incidence and mortality by detecting and removing advanced precancerous lesions, as well as by detecting cancerous lesions at an early stage. Chemoprevention has the potential to decrease the occurrence of cancer, as well as delay its onset. Numerous agents, such as metabolic agents, vitamin and minerals, non-steroidal anti-inflammatory (NSAIDs) drugs or aspirin, have been reported for CRC chemoprevention.^2^ The latter is likely the most promising agent that will be able to meet this aim. Aspirin, also known as acetylsalicylic acid (ASA), is primarily known for its analgesic, antipyretic actions but also as an agent for cardiovascular prophylaxis. It has been known for several years as a protective factor against cancer development, especially CRC. However, chemoprevention by aspirin remains controversial. This review closely details the mechanisms of action by which aspirin exerts its anti-tumor effects, as well as the primary clinical results of chemoprevention on neoplastic colorectal lesions. It also provides an overview of the biomarkers most likely to predict aspirin efficacy.

## I. Actions of aspirin on CRC tumourigenesis

**Figure 1.**
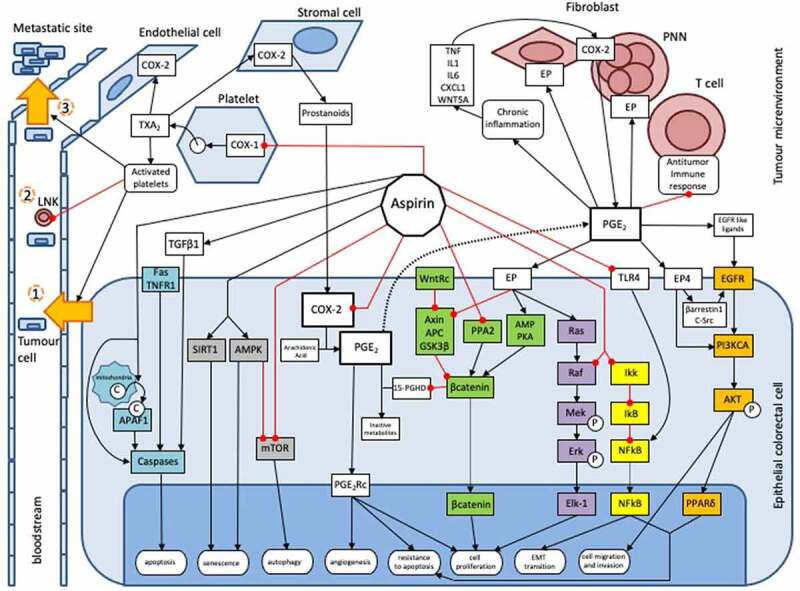
Actions of aspirin on colorectal tumourigenesis. This inhibitory action occurs through direct mechanisms on tumor cells, either by inhibiting the synthesis of prostaglandins and associated intracellular pathways, such as WNT (green), EGFR/PIK3CA/AKT/PPARδ (Orange) and Ras/Raf/MAP kinase (MEK)/ERK pathways (purple), or through an inhibition on prostaglandin-independent pathways, as NFkB (yellow), AMPK/mTOR (gray) and the apoptosis pathways (blue). Aspirin also exerts an anti-cancer action in an indirect way through its inhibitory action on various actors of the tumor microenvironment, such as platelets or the anti-inflammatory immune system. Due to this wealth in the multiple inhibitory mechanisms involved, aspirin is capable of inhibiting many properties of neoplastic cells, such as their antiapoptotic capacity or their potential for migration, invasion, proliferation, metastatic dissemination, etc.

Aspirin is a well-known protective factor against several cancers, especially CRC.^3^ However, its molecular mechanisms are incompletely understood. It exerts both direct mechanisms on CRC cells and indirect mechanisms on the tumor microenvironment (Figure 1).

AMP, adenosine monophosphate; AMPK, AMP-activated protein kinase; APAF1, apoptotic protease factor 1; C, cytochrome c; COX-1, cyclooxygenase 1; COX-2, cyclooxygenase 2; CRC, colorectal cancer; EGFR, epithelial growth factor receptor; 15-PGDH, 15 hydroperoxy prostaglandin dehydrogenase; P, phosphorylation; PGE_2_, prostaglandin E2; PGEM, PGE_2_ metabolites; PGE_2_Rc, PGE_2_ receptor; PIK3CA, phosphatidylinositol-3-kinase; PKA, protein kinase A; PPA2, phosphatase A2 protein; PPARδ, peroxisome proliferator-activated receptor-δ; SIRT1, Sirtuin 1; TGF-β1, transcription growth factor β1; TXA_2_, thromboxane A2.

### 1a. Direct mechanisms on epithelial cancer cell

#### Prostaglandins-mediated actions

Prostaglandins (PG) are bioactive lipids from the prostanoid family involved in several biological processes, including tumourigenesis. Biosynthesis of prostanoids begins with arachidonic acid, which is transformed by cyclooxygenase enzyme (COX) to an unstable intermediate, prostaglandin H2. Next, tissue-specific isomerases generate five different prostanoids: thromboxane A2 (TXA_2_), prostaglandin D2, prostaglandin E2 (PGE_2_), prostaglandin F2α and prostacyclin.^4^ Prostaglandins act through an autocrine or a paracrine effect by binding to specific transmembrane G protein-coupled receptors. PGE_2_ has four specific receptors, termed EP receptors 1, 2, 3 and 4. There are two different isoforms of COX enzyme: i) COX-1 exhibits ubiquitous expression and plays a role in platelet aggregation and gastric cytoprotection. Platelets express this isoform after maturation and produce TXA_2_. ii) COX-2 expression is only induced in certain tissues in response to inflammation, wound healing or neoplasia.^5^ Finally, the ubiquitous 15 hydroperoxy prostaglandin dehydrogenase enzyme (15-hydroperoxyPGDH) metabolizes PG to both inactive metabolites, 13,14-dihydro-15-keto-PGE2 (PGEM) and 13,14-dihydro-15-keto-PGF2α.^6^

Aspirin and others NSAIDs reduce PGE_2_ production through different mechanisms of COX enzyme inhibition.^7,8^ NSAIDs compete with arachidonic acid for reversible binding to a common docking site. Aspirin inhibits COX through irreversible selective acetylation of a critical serine residue. NSAIDs’ actions are thus reversible, whereas aspirin’s action is irreversible and requires new synthesis of COX enzymes.^5^ Low dose aspirin inhibits COX-1 (see section below on platelet inhibition), whereas daily high-dose aspirin is required to inhibit COX-2.^4,7^ A recent study conducted on animal models has interestingly underlines interest of an intermittent dosing-regimen of a low-dose aspirin treatment to prevent the occurrence of CRC, without increasing gastro-intestinal side effects.^9^

PGE_2_ is the prostanoid most involved in colorectal tumourigenesis.^10^ Several studies have reported that PGE_2_ and/or COX-2 levels are significantly higher in adenoma and CRC than in healthy tissue.^11,12^ In contrast, 15-hydroperoxyPGDH acts as a tumor suppressor protein, and its expression is downregulated in CRC cells, and restoration of 15-hydroperoxyPGDH expression exerts an antitumour effect.^6,13^ Many mechanisms have been proposed to explain the protumour role of PGE_2_. PGE_2_ acts directly on tumor epithelial cells by stimulating cellular proliferation and survival, as well as promoting migration and invasion of CRC cells. PGE_2_ induces cytoskeletal reorganization that changes cellular shape and facilitates motility of CRC cells.^10,14,15^

#### Action on intracellular pathways through PGE_2_ inhibition

PGE_2_ is implied in several intracellular pathways involved in colorectal tumorigenesis. The inhibitory action of aspirin on PGE_2_ will thus modulate colorectal tumorigenesis, through these pathways.

*The WNT signaling pathway* (in green on Figure 1)

WNT signaling comprises an important pathway involved in colorectal tumourigenesis. Briefly, when the WNT pathway is inactive, a destruction complex, formed by Axin, adenomatous polyposis coli protein (APC) and glycogen synthase kinase 3β (GSK3β), phosphorylates specific N-terminal residues in β-catenin, which is then recognized as part of a ubiquitin ligase complex that undergoes polyubiquitination, leading to proteasomal degradation. Protein phosphatase 2A (PP2A) decreases levels of phosphorylated β-catenin, preventing its degradation. Activation of the WNT pathway leads to cytoplasmic accumulation of β-catenin, then to nuclear translocation and transcription of target genes involved in cell proliferation.^16^ Several interaction loops exist between PGE_2_ and the WNT pathway. PGE_2_ stimulates the EP2 receptor, which binds to the Axin protein, inactivating the destruction complex.^17^ PGE_2_ increases cytoplasmic levels of β-catenin through a signaling pathway involving cyclic adenosine monophosphate (AMP) and protein kinase A (PKA).^18^ PGE_2_ also increases transcription of the peroxisome proliferator-activated receptor δ (PPARδ) gene.^19^ This gene is involved in colorectal tumourigenesis by promoting resistance to apoptosis and is one of the genes targeted by the WNT pathway.^20,21^ Furthermore, β-catenin inhibits 15-hydroperoxyPGDH expression through direct binding to its promoter, increasing PGE_2_ levels.^22^ Aspirin, in addition to its inhibitory role on PGE_2_ production, exerts an antitumour effect through another mechanism of negatively regulating the WNT pathway. Thus, aspirin causes inactivation of the phosphatase A2 (PPA2) protein through phosphorylation of its catalytic subunit and restores β-catenin degradation.^23–25^

*The EGFR/PIK3CA/AKT/PPARδ pathway* (in orange on Figure 1)

PGE_2_ stimulates the PIK3CA/AKT pathway primarily through EP4 receptor activation, which in turn either directly activates PIK3CA enzyme or EGFR transactivation through an intermediate β-arrestin-1–SRC complex or by stimulating secretion of EGF-like ligands.^19,26,27^ Aspirin inhibits PIK3CA/AKT pathway by reducing PGE_2_ levels and has a greater inhibitory effect on tumor growth in CRC cells with activating *PIK3CA* mutations.^28,29^

*The Ras/Raf/MAP kinase (MEK)/ERK pathway* (in purple on Figure 1)

The Ras/Raf/MEK/ERK cascade is an important pathway involved in colorectal tumourigenesis. PGE_2_ stimulates COX-2 expression through activation of the Ras/MEK/ERK pathway, forming a self-amplifying loop.^30^ Aspirin inhibits this pathway by increasing inhibitory phosphorylation of Raf, avoiding its interaction with Ras and hence, its activation.^31^

#### PGE_2_-independent actions

Several intracellular pathways involved in colorectal tumorigenesis are PGE_2_-independent. Aspirin also acts on these pathways, independently of PGE_2_ inhibition.

*The NFkB pathway* (in yellow on Figure 1)

NFkB is a transcription factor that stimulates expression of antiapoptotic genes and usually exists as a heterodimer complex bound in the cytoplasm by the inhibitor protein IKB. It has been reported that aspirin inhibits the NFkB pathway through binding to the IKB protein, belonging to the IKK complex. The IKB protein is normally phosphorylated by the IKK cellular kinase complex and then degraded by the proteasome machinery, enabling the release of sequestered NFkB in the cytoplasm and its translocation to the nucleus.^32^ Aspirin may thus cause retention of NFkB protein in the cytoplasm and repression of antiapoptotic genes transcription. However, this action should require very high doses of aspirin and has not been proven by *in vivo* studies.^33^ Furthermore, lipopolysaccharides from Gram-negative intestinal bacteria activate the epithelial-mesenchymal transition (EMT) of CRC cells through the stimulation of TLR-4 receptors with subsequent activation of the NFkB pathway. Aspirin has been shown to prevent EMT by inhibiting TLR-4 expression, which inhibits activation of the NFkB signaling pathway.^34^

*The AMPK/mTOR pathway* (in gray on Figure 1)

Sirtuin 1 (SIRT1) and AMP-activated protein kinase (AMPK) are two key regulators of cellular metabolism. Aspirin induces senescence of CRC cells by creating an energy imbalance through upregulation of SIRT1 and phospho-AMPK.^35^ The mammalian target of rapamycine (mTOR) pathway is involved in protein synthesis and cell growth. Aspirin inhibits mTOR both through a direct action and the activation of AMPK. This inhibition in turn induces autophagy in CRC cells.^36^

*The apoptosis pathway* (in blue on Figure 1)

There are two major apoptosis pathways, the mitochondrial (or intrinsic) pathway and the extrinsic pathway. In the first, cytochrome c, released by the mitochondria into cytosol, binds to apoptotic protease activating factor 1 (APAF1) to induce consecutive activation of caspases, the effector enzymes of the apoptosis pathways. In the second one, stimulation of death receptors, such as Fas or TNFR1, induces recruitment of the death inducing signaling complex, which activates caspase 8 secondarily and subsequently, downstream caspases. Aspirin induces apoptosis by stimulating the release of cytochrome c from mitochondria to cytosol, and by stimulating caspase 8 and caspase 3.^37,38^ Aspirin additionally increases secretion of TGF-β1, a cytokine involved in cell growth that has a key role in apoptosis regulation. TGF-β1 induces CRC cell apoptosis through downregulation of antiapoptotic Bcl-2 family members, upregulation of the pro-apoptotic factor Bax, and activation of caspase proteases.^39^

### 1b. Indirection mechanisms on tumor microenvironment

#### Action through platelet inhibition

As described above, the COX-2 enzyme plays an important role in colorectal carcinogenesis. COX-2 inhibition by aspirin is only temporary in nucleated cells because of de novo synthesis of COX-2; therefore, high-dose aspirin is required several times a day to directly inhibit COX-2 in epithelial cells. However, many observational studies have reported that low-dose aspirin given once daily decreases the incidence of CRC (see sections below). This finding could be due to the inhibitory effect of aspirin on platelets. COX-1 is the only isoform of the COX enzyme in mature platelets in which TXA_2_ is specifically produced. Due to lacking a nucleus, regeneration of COX-1 is not possible in platelets, and aspirin, despite its short half-life, exerts an irreversible action on COX-1. Platelets are activated in the tumor microenvironment after interaction with epithelial cancer cells.^40^ Activated platelets generate soluble growth and angiogenic factors, such as TXA_2_ and sphingosine 1 phosphate. TXA_2_ and others released factors stimulate recruitment and aggregation of additional platelets, and enhance COX-2 expression in stromal and endothelial cells in the tumor microenvironment.^33^ Then, stromal cells produce prostanoids and other growth factors that stimulate COX-2 expression in epithelial cancer cells. Thus, inhibition of platelet COX-1 by aspirin and COX-2 expression in return in epithelial cells is one of the inhibitory mechanisms of aspirin on platelet-mediated tumourigenesis.^33,41,42^

Activated platelets intervene at multiple stages of carcinogenesis. Firstly, activated platelet promotes cell proliferation through an upregulation of c-MYC.^43^ They also stimulate angiogenesis, and epithelial-mesenchymal transition of tumor cells. Thus, tumor cells become more invasive, and intravasate in blood vessels (step one in Figure 1).^44^ Then, platelets promote tumor cell survival in blood circulation by protecting them from natural killer (NK) cells. Secretion of TGF-β by activated platelets inhibits both the activation and function of NK cells.^45^ Transfer of major histocompatibility complex class I molecules from platelets to tumor cells might also allow them to evade lysis by NK cells (step two in Figure 1).^46^ Finally, platelets make extravasation and metastatic spread possible by promoting interactions between tumor cells and the vascular endothelium. CRC cells roll on activated endothelium, and this anchoring process is due to binding between CD44 on CRC cells and P-selectin on activated endothelial cells. Activated platelets interact with CRC cells in the bloodstream to form heteroaggregates that support attachment to the endothelium.^47^ Then, activated platelets release adenine nucleotides, which enhance endothelium permeability through their action on P2Y2 receptors of endothelial cells, stimulating extravasation of cancer cells (step three in Figure 1).^48^ Therefore, aspirin also exerts an antitumour effect through the inhibition of platelet activation.^33,49,50^

***Action on inflammatory and on immune cells*** (in red in Figure 1)

Chronic inflammation is an important hallmark of CRC since it contributes to its initiation, as well as its progression.^51^ Inflammation-induced cells, such as polymorphonuclear neutrophils and fibroblasts, produce pro-inflammatory cytokines and chemokines (TNF-α, IL1, IL6, CXCL1, and WNT5A) in the tumor microenvironment. These cytokines stimulate the production of PGE_2_ through increased activity of COX-2 of neutrophils and fibroblasts.^52^ Then, PGE_2_ activates EP2 receptors on fibroblasts and polymorphonuclear neutrophils in the tumor microenvironment, forming a positive feedback loop that exacerbates inflammation.^53^ Thus, the EP2 receptor could be an attractive target to inhibit inflammation-induced tumourigenesis, and several studies are currently being conducted to evaluate potent selective antagonists of this receptor.^54^ PGE_2_ induces massive recruitment of immune cells and modifies their functionality, particular in lymphocytes, by stimulation of Th1 differentiation and Th17 expansion.^55,56^ PGE_2_ also modifies local cytokine profiles through induction of IL-23 and inhibition of IL-12 expression to stimulate T helper 17 (Th17) cell expression.^57^ PGE_2_ also enhances dendritic cell migration through upregulation of CCR7, as well as their capacity to activate T cells.^58^

## II. Aspirin use to prevent or treat colorectal adenoma or cancer

### 2a. Aspirin use to prevent recurrence of colorectal adenoma

Most CRCs develop from precancerous adenomatous lesions through an adenoma–carcinoma sequence.^59^ Many observational studies and randomized controlled trials (RCTs) suggest a significant decrease in CRC incidence for regular aspirin users (see section below). Consequently, several studies have been conducted to assess whether aspirin prevents recurrence of adenoma (Table 1) and whether this antineoplastic effect was due to a decreased risk of adenoma formation or whether it intervened on the transformation of adenoma into cancerous lesions. Most of these studies, including several well-designed RCTs, shows that chemoprevention by aspirin reduces colorectal adenoma recurrence rate and decreases the rate of advanced adenoma recurrence, the number of recurrent adenomas and delays the time to adenoma recurrence.^60–62,64^ A recent meta-analysis found similar findings with a significant 20% reduction in risk in low-dose aspirin users (80 to 160 mg per day) compared to control patients (RR 0.80, CI 95% 0.70–0.92).^67^

**Table 1.** Primary studies concerning the prevention of colorectal adenoma recurrence by aspirin.

Authors [publication date] (ref)	Design	Primary endpoint	Inclusion criteria	Participants (n)	Duration of treatment	Aspirin Dose	Results [CI 95%]
Sandler et al. [2003] ^60^	Double-blinded RCT	Colorectal adenoma recurrence	Patients with previous Dukes A, B1 or B2 CRC, without aspirin treatment	517	≥ 1 year	325 mg	RR = 0.65 [0.46–0.91]
Baron et al. [2003]^61^	Double-blind RCT	Colorectal adenoma recurrence	Patients with a recent history of histologically documented colorectal adenoma	1 121	≥ 1 year	81 mg	RR = 0.81 [0.69–0.96]
						325 mg	RR = 0.96 [0.81–1.13]
Logan et al. [2008]^62^	Double-blinded RCT	Colorectal adenoma recurrence	Patients who had a colorectal adenoma removed in the 6 months before recruitment	853	3 year	300 mg	RR = 0.79 [0.63–0.99]
Benamouzig et al. [2012]^63^	Double-blind RCT	Colorectal adenoma recurrence	Aspirin-naive patients with a story of colorectal adenoma	After 1 y of follow-up: 238 After 4 y of follow-up: 185	4 year	160 or 300 mg	After 1 y of follow-up: RR = 0.73 [0.52–1.04] After 4 y of follow up: RR = 0.96 [0.75–1.22]
Ishikawa et al. [2014]^64^	Double-blinded RCT	Colorectal adenoma or adenocarcinoma recurrence	Asian patients with history of colorectal adenomas or adenocarcinomas (invasion limited to the mucosa) removed	311	2 year	100 mg	ORa = 0.60 [0.36–0.98]
Pommergaard et al. [2016]^65^	Double-blinded RCT	Colorectal adenoma recurrence	Patients with one or more sporadic colorectal adenoma removed	427	3 year	75 mg	OR = 0.95 [0.61–1.48]
Hull et al. [2018]^66^	Double-blinded RCT	Colorectal adenoma recurrence	Patients considered at high risk of adenomas (≥3 adenomas if at least one was ≥10 mm in diameter or ≥5 adenomas if there were <10 mm)	709	1 year	300 mg	RR = 0.99 [0.87–1.12]

CRC, colorectal cancer; NS, non-significant; ORa, odds ratio adjusted; RCT, randomized control trial; Ref, reference; RR, relative risk

However, two recent RCTs found no effect of aspirin chemoprevention on colorectal adenoma recurrence^65,66^ and one RCT showed that chemoprevention by aspirin reduced colorectal adenoma recurrence after 1 y, but not after 4 y, of follow-up.^63^ Some characteristics of the patients included in these latter trials might explain the lack of a protective effect of aspirin.

It has previously been reported that chemoprevention with daily low-dose aspirin reduced colorectal adenoma recurrence primarily in nonsmoking patients, while it was useless in patients who smoked.^64^ This could explain the negative results reported by Pomergaard et *al* due to the high proportion of smokers in this study.^65^ In the seAFOod Polyp Prevention trial, included patients corresponded to a high-risk population.^66^ The adenoma recurrence rate at 1 y was not significantly different between the experimental (300 mg aspirin per day) and control (placebo) groups (61% in both groups) and was substantially higher than those previously reported in similar trials. Secondary analysis showed that aspirin treatment reduced the total number of colorectal adenoma per participant.^66^ Therefore, the number of recurrent adenomas might be a more appropriate endpoint for evaluating the interest of chemoprevention by aspirin in this specific population.^68^

These findings suggest that daily low-dose aspirin decreases the risk of colorectal adenoma recurrence with a benefit that would appear to be reduced in smokers and high-risk patient subgroups. For this latter subgroup, further trials assessing specific experimental conditions (dosing and duration of treatment) and endpoints are warranted.

### 2b. Aspirin use to prevent CRC

Several observational studies have identified aspirin as a protective factor against CRC development (Table 2). For example, a Danish retrospective case-control study showed a significant reduction of CRC incidence in patients who were treated with daily low-dose aspirin for more than 5 y (odds ratio of 0.73 (CI 95% 0.54–0.99)).^69^ Another recent similar study in the UK also found a 34% decrease in the risk of developing CRC in patients with daily low-dose aspirin (RR 0.66, CI 95% 0.60–0.74).^71^ A meta-analysis of five RCTs showed a 24% decrease in the risk for developing CRC (HR 0.76, CI 95% 0.60–0.96). This risk was especially reduced for proximal colon cancers. Daily low-dose aspirin also showed a 40% reduction for the risk of CRC mortality (HR 0.60, CI 95% 0.42–0.86). However, the results of RCTs remain controversial. Recently, the ASPREE trial was conducted to assess the impact of low-dose aspirin on disability-free survival among older adults who did not have cardiovascular disease, dementia, or disability. Secondary analyses demonstrated that aspirin use was associated with a higher risk for cancer-related mortality (HR 1.31, CI 95% 1.10–1.56) and colorectal cancer-related mortality (HR = 1.77, CI 95% 1.02–3.06). The short median follow-up of 4.7 y and the inclusion of older patients could potentially explain this lack of benefit.^75^

**Table 2.** Primary studies concerning primary prevention by aspirin of colorectal cancer.

Authors [publication date] (ref)	Design	Primary endpoint	Inclusion criteria	Participants (n)	Follow-up	Aspirin dose	Results [CI 95%]
Friis et al. [2015]^69^	Case-control study	CRC	Incident cases of CRC diagnosed in North of Denmark between 1994 and 2001	10 280 cases 102 800 controls	NA	Low dose of aspirin (75, 100 or 150 mg)	OR = 0.73 [0.54–0.99]
Rothwell et al. [2010]^70^	Meta-analysis of 5 RCT	CRC incidence and CRC-specific mortality	Patients with a risk of cardiovascular events. Patients with history of gastroduodenal ulcer or myocardial infarction were excluded.	14 033	≥ 18 year	2 studies with low dose (75 mg) 2 studies with high dose (≥ 300 mg) One study involving different doses of aspirin	CRC incidence: HR = 0.76 [0.60–0.96] CRC-specific mortality: HR = 0.65 [0.48–0.88]
Garcia Rodriguez et al. [2017]^71^	Case-control study	CRC	Patients from a UK primary database, aged 40 to 84 y between 2000 and 2009	3 033 cases 10 000 controls	NA	Low dose of aspirin (75 to 300 mg)	RR = 0.66 [0.60–0.74]
Stürmer et al. [1998]^72^	RCT	CRC	Healthy male physicians living in the US, aged 40 to 84 in 1982.	22 071	12 y (1982–1995)	High dose of aspirin (325 mg)	RR = 1.03 [0.83–1.28]
Cook et al. [2005]^73^	RCT	CRC	US Women aged at least 45 y and initially without history of cancer, cardiovascular diseases, or other major chronic illness	39 876	10 y (1993–2004)	Low dose of aspirin (100 mg)	RR = 0.97 [0.77–1.24]
Cook et al. [2013]^74^	Follow-up of a cohort from a RCT	CRC	33 682 women among the 39 876 from the Women’s Health Study continued observational follow-up until March 2012	33 682	≥ 10 year	Low dose of aspirin (100 mg)	HR = 0.80 [0.67–0.97]
McNeil et al. [2018]^75^	RCT	Cancer-related death	Patients from Australia or USA, who were 65 aged or older, from 2010 through 2014, without cardiovascular disease, dementia, or disability.	19 114	Median = 4.7 year	Low dose of aspirin (100 mg)	HR = 1.31 [1.10–1.56]

CI, confidence interval; CRC, colorectal cancer; HR, hazard ratio; NA, not available; OR, odds ratio; RCT, randomized control trial; Ref, reference; RR, relative risk

However, the studies by Rothwell et *al* and McNeil et *al* were not initially designed to assess CRC prevention.^70,75^ Specific RCTs have been conducted to demonstrate a cause and effect relationship and have measured the occurrence of CRC as a primary endpoint. Two large RCTs, the Physicians’ Health Study (PHS) (n = 22,071) and the Women’s Health Study (WHS) (n = 39,876), failed to demonstrate a protective effect of low-dose aspirin on CRC development in men and women, respectively.^72,73^ Indeed, no difference in favor of the experimental arm was found for either trial at the planned end of these studies, despite a follow-up of 12 y in the PHS and 9 y in the WHS.^72,73^ However, in the WHS, an extended follow-up of 18 y after randomization showed a significant association between chemoprevention by aspirin and decreased incidence of CRC (HR = 0.80; 95% CI 0.67–0.97).^74^ This long latent period to observe a benefit suggests that chemoprevention by aspirin act in the early stages of tumorigenesis.

All of these studies support that low-dose aspirin decreases long-term risk (more than 10 y) of CRC, as well as its related mortality, in populations over 50 y of age and who are at high cardiovascular risk. All these scientific studies have led to recent publication of the United States Preventive Service Task Force recommendations. Aspirin is currently recommended for primary prevention of cardiovascular events and CRC in patients aged 50 to 59 with a 10-y risk of cardiovascular event greater than 10%. This may also be the case for some patients aged 60 to 69 y old with a 10-y risk of cardiovascular event greater than 10% without significant risk of bleeding and with a life expectancy of at least 10 y. Aspirin is not yet recommended in primary prevention for only the risk of CRC.^76^

### 2c. Aspirin use in patients with genetic predisposition to CRC

Patients with familial adenomatous polyposis (FAP) or hereditary non-polyposis colorectal cancer (HNPCC) are considered to have a very high risk for developing CRC. Interest in chemoprevention by aspirin has been studied in this specific population. The CAPP1 (Colorectal Adenoma/Carcinoma Prevention Programme 1) study, comparing 600 mg aspirin treatment to placebo in patients with FAP aged 10 to 21 did not find a significant difference in colorectal polyp burden (RR = 0.77, CI 95% [0.54–1.10]). However, this trial showed that aspirin treatment significantly reduced the size of the largest polyp.^77^ The CAPP2 trial compared a 2-y treatment with aspirin at 600 mg to placebo in patients with Lynch syndrome.^78^ Results from the 10-y follow-up of this cohort have shown that aspirin significantly decreased the risk of CRC (adjusted HR = 0.65, 95% CI [0.43–0.97]) without increasing risk of serious adverse events. However, no difference was seen concerning the risk of other cancers of the Lynch syndrome spectrum (adjusted HR = 0.94, 95% CI [0.59–1.50]).^79^ Thus, daily chemoprevention with 600 mg of aspirin during at least 2 y must be considered in young patients with Lynch syndrome. Current trials in Lynch syndrome patient populations aim to identify the optimal duration of aspirin treatment, as well as the possibility of decreasing the dosage.^80,81^ Thus, the CAPP3 phase 3 trial (NCT02497820) compares daily dose of 600 mg versus 300 mg versus 100 mg aspirin treatment (primary outcome: number of new primary mismatch repair deficient cancers at 5 y), while the French AAS-Lynch trial (NCT02813824) compares a daily aspirin dose of 100 mg versus 300 mg versus placebo (primary outcome: number of patients with at least one colorectal adenoma at 4 y).

### 2d. Aspirin use to treat CRC

Evidence has also been reported concerning the interest of aspirin after CRC occurrence.^82^ Two aspects should be considered, depending on whether the tumor burden has been removed or is still present. Patients treated with daily low-dose aspirin are less likely to develop advanced stage CRC.^71^ This suggests that aspirin affects the progression of established CRC.^71^ This treatment could also potentiate the effect of conventional therapies since a retrospective study reported that long-term use of 100 mg aspirin in combination with neoadjuvant chemoradiotherapy increased the rate of downstaging (67.6% vs 43.6%, p = .01) and the pathological response rate (46% vs 19%; p < .001) in patients with rectal cancer.^83^ Daily low-dose aspirin treatment also decreases the risk of metastatic progression. A meta-analysis of five major studies comparing aspirin versus placebo in cardiovascular disease prevention showed that patients who develop adenocarcinoma are less likely to experience metastatic progression if they are treated with aspirin (HR 0.54, CI 95% 0.38–0.77, p = .0007). In patients with localized CRC, follow-up showed that the risk of metastatic progression was significantly lower in the aspirin group than in the control group (HR 0.26, CI 95% 0.11–0.57, p = .0008).^84^ Daily low-dose aspirin reduces the number of circulating tumor cells and avoids the epithelial mesenchymal transition in patients with metastatic CRC.^85^

In addition to this effect on tumor progression and downstaging, several studies have shown that aspirin use after CRC diagnosis was associated with reduced CRC-specific^82,86–88^ and overall mortality.^82,88–90^ However, two observational studies found no association between aspirin and either CRC-specific or overall survival in patients with CRC (Table 3). ,^91^,^92^

**Table 3.** Primary studies assessing aspirin as a treatment of colorectal cancer.

Authors [publication date] (ref)	Design	Primary endpoint	Inclusion criteria	Aspirin dose	Participants (n)	Results [CI 95%]
Chan et al. [2009]^82^	Prospective cohort study	All-cause mortality and CRC-specific mortality	Patients with a stage I to III* CRC	80 or 325 mg	1 279	All-cause mortality for post-diagnosis aspirin use: Adjusted HR = 0.79 [0.65–0.97] CRC-specific mortality for post-diagnosis aspirin use: Adjusted HR = 0.71 [0.53–0.95]
Bastiaannet et al. [2012]^87^	Observational study	All-cause mortality	Patients with a stage I to IV* CRC (16% of stage IV)	30 mg (5%) 80 mg (95%)	4 481	Adjusted RR = 0.77 [0.63–0.95]
Li et al. [2015]^86^	Meta-analysis	Overall mortality and CRC-specific mortality	Patients with a stage I to IV* CRC	NR	34 933	Overall mortality for post-diagnosis aspirin use: HR = 0.84 [0.75–0.94] CRC-specific mortality for post-diagnosis aspirin use: HR = 0.77 [0.52–1.14]
McCowan et al. [2013]^88^	Cohort study	All-cause mortality and CRC-specific mortality	Patients with a stage A to D** CRC	NR	2 990	All-cause mortality for post-diagnosis aspirin use: Adjusted HR = 0.67 [0.57–0.79] CRC specific mortality for post-diagnosis aspirin use: Adjusted HR = 0.58 [0.45–0.75]
Goh et al. [2015]^89^	Observational Study	CRC-specific mortality	Patients with a stage I to III* CRC	NR	726	CRC-specific mortality for post-diagnosis aspirin use: Adjusted HR = 0.38 [0.17–0.84]
Bains et al. [2016]^90^	Retrospective cohort study	Overall survival and CRC-specific survival	Patients with a stage I to IV* CRC (16,2% of stage IV)	75 or 160 mg	23 162	Overall survival for post-diagnosis aspirin use: Adjusted HR = 0.95 [0.90–1.01] CRC-specific survival for post-diagnosis aspirin use: Adjusted HR = 0.85 [0.79–0.92]
Cardwell et al. [2014]^91^	Observational Study	All-cause mortality and CRC-specific mortality	Patients with a stage I to IV* CRC (12% of stage IV)	25 mg (0.3%) 75 mg (98.5%) ≥ 300 mg (1.2%)	4 794	All-cause mortality for post-diagnosis aspirin use: Adjusted OR = 1.06 [0.94–1.19] CRC-specific mortality for post-diagnosis aspirin use: Adjusted OR = 1.06 [0.92–1.24]
Gray et al. [2018]^92^	Cohort study	CRC-specific mortality	Patients with a stage A to C** CRC	75 mg	8 391	Post-diagnostic low dose aspirin use: Adjusted HR = 1.17 [1.00–1.36] Pre-diagnostic low dose aspirin use: Adjusted HR = 0.96 [0.88–1.05]

CI, confidence interval; CRC, colorectal cancer; HR, hazard ratio; NR, not reported; OR, odds ratio; Ref, reference; RR, relative risk; *stages from the UICC TNM classification, **stages from the Dukes staging system

Regarding this evidence, several RCTs assessing aspirin as an adjuvant therapy in CRC are currently being conducted. The ASCOLT trial (NCT00565708) aims to evaluate whether adjuvant treatment with 200 mg aspirin improves survival in patients with stage III and high-risk stage II CRC compared to placebo.^93^ ASPIK is a French trial assessing 100 mg aspirin versus placebo in a specific population of patients with *PIK3CA*-mutated stage III or high-risk stage II tumors (See predictive biomarkers section).^94^ The APREMEC trial (NCT02607072) is testing an adjuvant treatment with 100 mg or 200 mg aspirin compared to placebo in patients who underwent CRC resection.^95^ The ADD-ASPIRIN trial (ISRCTN74358648) is assessing whether daily 100 mg or 300 mg aspirin use after standard cancer therapy prevents recurrence and prolongs survival in patients with early-stage solid tumors, including those of CRC.^96^

## III. Predictive biomarkers indicating the efficacy of aspirin

The benefits of aspirin require long-term administration whose side effects are rare but may sometimes be life threatening. This highlights the need to develop a precision medicine strategy to identify patients who are most likely to benefit from prophylactic low-dose aspirin treatment.

### 3a. Genetic biomarkers

Constitutional biomarkers are present physiologically throughout the body, as opposed to the somatic markers present or produced specifically after the development of cancer. Several studies have shown that chemoprevention by aspirin differed according to certain genetic variations. Indeed, specific single nucleotide polymorphisms (SNPs) have been associated with a lower risk of CRC in response to regular aspirin use (Table 4). ,^97^,^98^,^99^,^100^,^101^ These genetic markers have so far been primarily described in situations of primary prevention. Validation of such genetic markers could be helpful for selecting responder patients to long-term aspirin use and could represent the first step of a chemoprevention strategy adapted to individual genetic characteristics.

**Table 4.** Studies on germline genetic biomarkers associated with efficacy of aspirin chemoprevention on colorectal adenoma or cancer occurrence.

Authors [publication date] (ref)	Design	Aspirin dose	Primary endpoint	Participants (n)	Germline biomarkers (localization)	Results [CI 95%]
Nan et al. [2015]^97^	Case control study	NA	CRC	8634	rs2965667 (ch12)	TT: OR = 0.66 [0.61–0.70] TA or AA: OR = 1.89 [1.27–2.81]
					rs16973225 (ch15)	AA: OR = 0.66 [0.62–0.71] AC or CC: OR = 0.97 [0.78–1.20]
Nan et al. [2013]^98^	Case control study	NA	CRC	840	rs6983267 (ch8)	T: Adjusted OR = 0.83 [0.74–0.84]
Resler et al. [2014]^99^	Case control study	NA	CRC	1621	rs2920421 (*ALOX12* gene)	GA: OR = 0.60 [0.45–0.80]
Hubner et al. [2008]^100^	Meta-analysis of 3 studies	81 or 325 mg	Adenoma	2207	Homozygous ODC316AA Genotype (*ODC316* Gene)	RR = 0.52 [0.29–0.91]
Barry et al. [2011]^101^	Cohort study	81 or 325 mg	Adenoma	792	rs2430420 (*ODC* gene)	GG: RR = 0.68 [0.50–0.94]
					rs28362380 (*ODC* gene)	TT: RR = 0.75 [0.61–0.92]

Ch, chromosome; CI, confidence interval; CRC, colorectal cancer; NA, not available; OR, odds ratio; RCT, randomized control trial; Ref, reference; RR, relative risk

### 3b. Somatic biomarkers

We previously stated that long-term aspirin use reduced the risk of tumor progression, as well as the recurrence rate, and improved overall survival after resection. This benefit seems to differ depending on the molecular tumor characteristics and the pathways involved in carcinogenesis.

#### Prostanoids pathway

Aspirin exerts its biological effects primarily through binding to the COX enzyme with a consequent reduction in the functionality of the enzyme and in turn, the resultant production of downstream mediators, such as PGE_2_. It is therefore natural to imagine that a preexisting imbalance in the concentration of mediators involved in this pathway could modify the effects of aspirin. A recent meta-analysis reported that COX-2 expression highlighted in the tumor tissue was significantly associated with a decreased risk of death in postdiagnosis aspirin users (HR 0.65, CI 95% 0.50–0.85).^86^ Gray et *al* also confirmed these findings, showing that COX-2 expression was associated with improved overall survival in aspirin users (HR = 0.64, 95% CI 0.42–0.98) compared to non-users (HR = 1.28, 95% CI 0.80–2.03).^102^ Prostaglandin metabolite (PGE-M) could also be an interesting biomarker since several studies have found that advanced and/or multiple colorectal adenomas were significantly associated with higher levels of PGE-M.^103,104^ A prospective case–control study nested within the Nurses’ Health Study found that regular aspirin use reduced the risk for adenoma only in women with high levels of PGE-M compared to those with low levels (multivariate OR = 0.61; 95% CI, 0.43–0.87).^104^ Finally, the 15-hydroperoxyPGDH enzyme, which catalyses prostaglandin degradation, could also be a major factor in determining aspirin efficacy. Another case-control study conducted within the Nurses’ Health Study and the Health Professionals Follow-Up Study showed that long-term aspirin users, a lower risk of colorectal cancer was found only in patients with high levels of 15-hydroperoxyPGDH (multivariable HR = 0.49; 95% CI, 0.34 to 0.71).^105^ This biomarker could also be interesting, as its levels appear stable over time and seem not to be altered by aspirin exposition.^106^

#### Ras/Raf/MAP and EGFR/PI3KCA/AKT pathways

*RAS* and *BRAF* mutations are mutually exclusive and correspond to common molecular alterations in colorectal carcinogenesis. Aspirin’s effects seem more pronounced in wild-type tumors for both genes. A retrospective study found that regular aspirin use was associated with reduced risk of *BRAF* wild-type CRC but not with *BRAF*-mutated CRC.^107^ Another study showed that postdiagnostic NSAID use, including aspirin, improved overall survival only in patients with *KRAS* wild-type tumors (HR = 0.60, 95% CI [0.46–0.80]) compared to *KRAS*-mutated tumors (HR = 1.24, 95% CI [0.78–1.96]).^108^ In this study, *BRAF* mutation status was not predictive of postdiagnostic aspirin efficacy. *PIK3CA* mutations occur in 11–15% of CRCs and could be a predictive biomarker of aspirin efficacy in patients with CRC.^86^ Subgroup analysis suggested that aspirin use may reduce CRC recurrence rate after resection and improve CRC-specific survival in patients with *PIK3CA*-mutated CRCs.^109,110^ However, the results from four additional recent retrospective studies were inconsistent with this observation (Table 5).^102^,^109^,^110^,^111^,^112^,^113^ A meta-analysis of the first three studies^109,110,111^ showed an association between aspirin and a reduced risk of death in patients with *PIK3CA*-mutated CRC (HR = 0.58, 95% CI [0.37–0.90]).^86^ Given these discordant results, a French RCT (PRODIGE 50 – ASPIK) is currently being conducted. This trial compares adjuvant treatment with aspirin (100 mg per day) to placebo in patients with *PIK3CA*-mutated tumors who underwent surgery for stage III or high-risk stage II CRC. The primary endpoint corresponds to the 3 y disease-free survival rate.^94^

**Table 5.** Retrospective studies assessing the efficacy of aspirin in patients with *PIK3CA* mutation who underwent colorectal cancer resection.

Authors [publication date] (ref)	Design	Primary endpoint	Inclusion criteria	Aspirin dose	Participants (n) (Total/aspirin users)	Number of patients with *PIK3CA* mutated tumor	Results [CI 95%]
Liao X et al. [2012]^109^	Cohort study	Overall survival and CRC-specific survival	Patients who undergone a surgery for a stage I to IV* CRC (7% of stage IV)	81 or 325 mg	964/403	161	Overall survival: HR = 0.54 [0.31–0.94] CRC-specific survival: HR = 0.18 [0.06–0.61]
Domingo E et al. [2013]^110^	Cohort study	Recurrence-free survival	Patients who undergone a surgery for a stage II to III* CRC	≤ 100 mg	896/125	104	HR = 0.11 [0.001–0.832]
Reimers MS et al. [2014]^111^	Cohort study	Overall survival	Patients who undergone a surgery for a stage I to IV* CRC (16% of stage IV)	NA	663/174	100	HR = 0.73 [0.33–1.63]
Kothari N et al. [2015]^112^	Cohort study	Overall survival and CRC-specific survival	Patients who undergone a surgery for a stage I to IV* CRC (24% of stage IV)	≥ 75 mg	1487/387	185	Overall survival: HR = 0.96 [0.58–1.57] CRC-specific survival: HR = 0.60 [0.34–1.16]
Gray RT et al. [2017]^102^	Cohort study	Overall survival and CRC-specific survival	Patients who undergone a surgery for a stage II or III CRC	75 mg	740/146	109	Overall survival: HR = 0.90 [0.39–2.05] CRC-specific survival: HR = 0.74 [0.24–2.28]
Murphy C et al. [2017]^113^	Cohort study	Recurrence-free survival	Patients who undergone a surgery for a stage II CRC	≥ 75 mg	488/95	70	HR = 0.45 [0.06–3.70]

CI, confidence interval; CRC, colorectal cancer; HR, hazard ratio; NA, not available; Ref, reference; *stages from the UICC TNM classification

## IV. Aspirin and CRC screening

CRC chemoprevention by long-term use of aspirin raises the question of its association with CRC screening. Fecal immunochemical testing (FIT) is the most often used method for CRC screening. This test is based on the identification of human hemoglobin in a stool sample. It has been proven to reduce CRC mortality.^114^ Aspirin treatment may affect FIT results in inconsistent ways. On the one hand, aspirin could increase bleeding from colorectal adenoma or carcinoma, enhancing FIT sensitivity; but on the other hand, aspirin could increase physiological blood loss from colonic mucosa or from non-tumor lesions, decreasing FIT specificity and predictive positive value. Concerning FIT sensibility, Brenner et *al* showed that a single aspirin dose given before the FIT did not increase its sensitivity in patients not taking antithrombotic drugs.^115^ Concerning FIT predictive positive values, a large Norwegian cohort study found that regular aspirin use was associated with lower positive predictive value of FIT for detection of CRC and advanced adenomas.^116^ However, a recent meta-analysis showed that aspirin treatment did not affect FIT accuracy.^117^ According to these studies, current guidelines recommend to continue aspirin treatment during the FIT.^118^

## V. Risks of long-term aspirin treatment

Long-term aspirin use is associated with the occurrence of adverse effects, primarily including an increased risk of bleeding events.^119^ A meta-analysis involving 35 RCTs (low-dose aspirin alone) and 87,581 individuals confirmed this increase of any bleeds, any major gastrointestinal bleeds (GI) and any GI bleeds with hazard ratios of 1.54 (95% CI 1.36–1.74), 1.55 (95% CI 1.27–1.90) and 1.31 (95% CI 1.21–1.42), respectively.^120^ However, there was no meaningful difference concerning the risk for fatal bleeds (OR = 1.22, 95% CI [0.78–1.89]). Furthermore, there is a relationship between the dose of aspirin or increasing age and the risk of bleeding events.^121^ Prophylaxis for bleeding may be associated with co‐administration of a proton pump inhibitor, especially in cases of aspirin use combined with another platelet aggregation inhibitor or anticoagulant, since it has been reported to reduce the risk of major GI bleeding (OR = 0.34, 95% CI [0.21–0.57]).^120^

## Conclusions

Daily low-dose aspirin treatment is a protective factor against the occurrence of CRC. Regarding the scientific evidence, US authorities published recommendations concerning chemoprevention of CRC using aspirin in patients aged 50 to 59 with a 10-y risk of cardiovascular events greater than 10%. In patients with CRC, aspirin use seems to reduce the risk of tumor progression and the recurrence rate after resection and may also increase overall survival. The knowledge of inhibitory mechanisms induced by aspirin on colorectal carcinogenesis has made it possible to identify predictive biomarkers for CRC chemoprevention. This effect seems to be particularly interesting in tumors with *PIK3CA* mutations and/or involvement of the prostanoid pathway.

## Supplementary Material

Supplemental Material

## Data Availability

Data sharing is not applicable to this article as no new data were created or analyzed in this study.

## References

[1] Bray F, Ferlay J, Soerjomataram I, Siegel RL, Torre LA, Jemal A. Global cancer statistics 2018: GLOBOCAN estimates of incidence and mortality worldwide for 36 cancers in 185 countries. CA Cancer J Clin. 2018;68(6):394–424. doi:10.3322/caac.21492. 30207593

[2] Katona BW, Weiss JM. Chemoprevention of colorectal cancer. Gastroenterology. 2020;158(2):368–388. doi:10.1053/j.gastro.2019.06.047. 31563626 PMC6981249

[3] Qiao Y, Yang T, Gan Y, Li W, Wang C, Gong Y, Lu Z. Associations between aspirin use and the risk of cancers: a meta-analysis of observational studies. BMC Cancer. 2018;18 doi:10.1186/s12885-018-4156-5. PMC5851082 29534696

[4] Patrono C, García Rodríguez LA, Landolfi R, Baigent C. Low-dose aspirin for the prevention of atherothrombosis. N Engl J Med. 2005;353(22):2373–2383. doi:10.1056/NEJMra052717. 16319386

[5] Thun MJ, Jacobs EJ, Patrono C. The role of aspirin in cancer prevention. Nat Rev Clin Oncol. 2012;9(5):259–267. doi:10.1038/nrclinonc.2011.199. 22473097

[6] Yan M, Rerko RM, Platzer P, Dawson D, Willis J, Tong M, Lawrence E, Lutterbaugh J, Lu S, Willson JKV, et al. 15-Hydroxyprostaglandin dehydrogenase, a COX-2 oncogene antagonist, is a TGF-β-induced suppressor of human gastrointestinal cancers. Proc Natl Acad Sci U S A. 2004;101(50):17468–17473. doi:10.1073/pnas.0406142101. 15574495 PMC536023

[7] Chan AT, Ogino S, Fuchs CS. Aspirin and the risk of colorectal cancer in relation to the expression of COX-2. N Engl J Med. 2007;356(21):2131–2142. doi:10.1056/NEJMoa067208. 17522398

[8] Kuo C-N, Pan -J-J, Huang Y-W, Tsai H-J, Chang W-C. Association between nonsteroidal anti-inflammatory drugs and colorectal cancer: a population-based case–control study. Cancer Epidemiol Biomarkers Prev. 2018;27(7):737–745. doi:10.1158/1055-9965.EPI-17-0876. 29695380

[9] Mohammed A, Janakiram NB, Madka V, Zhang Y, Singh A, Biddick L, Li Q, Lightfoot S, Steele VE, Lubet RA, et al. Intermittent dosing regimens of aspirin and naproxen inhibit azoxymethane-induced colon adenoma progression to adenocarcinoma and invasive carcinoma. Cancer Prevention Research (Philadelphia, Pa. 2019;12(11):751. doi:10.1158/1940-6207.CAPR-19-0312. PMC6849393 31530543

[10] Wang D, DuBois RN. Eicosanoids and cancer. Nat Rev Cancer. 2010;10(3):181–193. doi:10.1038/nrc2809. 20168319 PMC2898136

[11] Eberhart CE, Coffey RJ, Radhika A, Giardiello FM, Ferrenbach S, DuBois RN. Up-regulation of cyclooxygenase 2 gene expression in human colorectal adenomas and adenocarcinomas. Gastroenterology. 1994;107(4):1183–1188. doi:10.1016/0016-5085(94)90246-1. 7926468

[12] Zhang H, Sun X-F. Overexpression of cyclooxygenase-2 correlates with advanced stages of colorectal cancer. Am J Gastroenterol. 2002;97(4):1037–1041. doi:10.1111/j.1572-0241.2002.05625.x. 12003384

[13] Myung S-J, Rerko RM, Yan M, Platzer P, Guda K, Dotson A, Lawrence E, Dannenberg AJ, Lovgren AK, Luo G, et al. 15-Hydroxyprostaglandin dehydrogenase is an in vivo suppressor of colon tumorigenesis. Proc Natl Acad Sci U S A. 2006;103(32):12098–12102. doi:10.1073/pnas.0603235103. 16880406 PMC1567703

[14] Sheng H, Shao J, Washington MK, DuBois RN. Prostaglandin E2 increases growth and motility of colorectal carcinoma cells. J Biol Chem. 2001;276(21):18075–18081. doi:10.1074/jbc.M009689200. 11278548

[15] Wang D, DuBois RN. An inflammatory mediator, prostaglandin E2, in colorectal cancer. Cancer J. 2013;19(6):502–510. doi:10.1097/PPO.0000000000000003. 24270349 PMC4797645

[16] Fearon ER. Molecular genetics of colorectal cancer. Annu Rev Pathol. 2011;6(1):479–507. doi:10.1146/annurev-pathol-011110-130235. 21090969

[17] Castellone MD, Teramoto H, Williams BO, Druey KM, Gutkind JS. Prostaglandin E2 promotes colon cancer cell growth through a G s -Axin-ß-catenin signaling axis. Science. 2005;310(5753):1504–1510. doi:10.1126/science.1116221. 16293724

[18] Goessling W, North TE, Loewer S, Lord AM, Lee S, Stoick-Cooper CL, Weidinger G, Puder M, Daley GQ, Moon RT, et al. Genetic interaction of PGE2 and Wnt signaling regulates developmental specification of stem cells and regeneration. Cell. 2009;136(6):1136–1147. doi:10.1016/j.cell.2009.01.015. 19303855 PMC2692708

[19] Wang D, Wang H, Shi Q, Katkuri S, Walhi W, Desvergne B, Das SK, Dey SK, DuBois RN. Prostaglandin E2 promotes colorectal adenoma growth via transactivation of the nuclear peroxisome proliferator-activated receptor δ. Cancer Cell. 2004;6(3):285–295. doi:10.1016/j.ccr.2004.08.011. 15380519

[20] Gupta RA, Wang D, Katkuri S, Wang H, Dey SK, DuBois RN. Activation of nuclear hormone receptor peroxisome proliferator–activated receptor-δ accelerates intestinal adenoma growth. Nat Med. 2004;10(3):245–247. doi:10.1038/nm993. 14758356

[21] He T-C, Chan TA, Vogelstein B, Kinzler KW. PPARδ is an APC-regulated target of nonsteroidal anti-inflammatory drugs. Cell. 1999;99(3):335–345. doi:10.1016/S0092-8674(00)81664-5. 10555149 PMC3779681

[22] Smartt HJM, Greenhough A, Ordóñez-Morán P, Talero E, Cherry CA, Wallam CA, Parry L, Al Kharusi M, Roberts HR, Mariadason JM, et al. β-catenin represses expression of the tumour suppressor 15-prostaglandin dehydrogenase in the normal intestinal epithelium and colorectal tumour cells. Gut. 2012;61(9):1306–1314. doi:10.1136/gutjnl-2011-300817. 22082586

[23] Gala MK, Chan AT. Molecular pathways: aspirin and wnt signaling—a molecularly targeted approach to cancer prevention and treatment. Clin Cancer Res. 2015;21(7):1543–1548. doi:10.1158/1078-0432.CCR-14-0877. 25501125 PMC4383688

[24] Drew DA, Cao Y, Chan AT. Aspirin and colorectal cancer: the promise of precision chemoprevention. Nat Rev Cancer. 2016;16(3):173–186. doi:10.1038/nrc.2016.4. 26868177 PMC6741347

[25] Bos CL, Kodach LL, van den Brink GR, Diks SH, van Santen MM, Richel DJ, Peppelenbosch MP, Hardwick JCH. Effect of aspirin on the Wnt/β-catenin pathway is mediated via protein phosphatase 2A. Oncogene. 2006;25(49):6447–6456. doi:10.1038/sj.onc.1209658. 16878161

[26] Buchanan FG, Gorden DL, Matta P, Shi Q, Matrisian LM, DuBois RN. Role of β-arrestin 1 in the metastatic progression of colorectal cancer. Proc Natl Acad Sci U S A. 2006;103(5):1492–1497. doi:10.1073/pnas.0510562103. 16432186 PMC1360588

[27] Buchanan FG, Wang D, Bargiacchi F, DuBois RN. Prostaglandin E2 regulates cell migration via the intracellular activation of the epidermal growth factor receptor. J Biol Chem. 2003;278(37):35451–35457. doi:10.1074/jbc.M302474200. 12824187

[28] Zumwalt TJ, Wodarz D, Komarova NL, Toden S, Turner J, Cardenas J, Burn J, Chan AT, Boland CR, Goel A. Aspirin-induced chemoprevention and response kinetics are enhanced by PIK3CA mutations in colorectal cancer cells. Cancer Prev Res (Phila). 2017;10(3):208–218. doi:10.1158/1940-6207.CAPR-16-0175. 28154202 PMC5337164

[29] Gu M, Nishihara R, Chen Y, Li W, Shi Y, Masugi Y, Hamada T, Kosumi K, Liu L, da Silva A, et al. Aspirin exerts high anti-cancer activity in PIK3CA -mutant colon cancer cells. Oncotarget. 2017;8(50):87379–87389. doi:10.18632/oncotarget.20972. 29152088 PMC5675640

[30] Wang D, Buchanan FG, Wang H, Dey SK, DuBois RN. Prostaglandin E2 enhances intestinal adenoma growth via activation of the Ras-mitogen-activated protein kinase cascade. Cancer Res. 2005;65(5):1822–1829. doi:10.1158/0008-5472.CAN-04-3671. 15753380

[31] Pan M-R, Chang H-C, Hung W-C. Non-steroidal anti-inflammatory drugs suppress the ERK signaling pathway via block of Ras/c-Raf interaction and activation of MAP kinase phosphatases. Cell Signal. 2008;20(6):1134–1141. doi:10.1016/j.cellsig.2008.02.004. 18374541

[32] Yin M-J, Yamamoto Y, Gaynor RB. The anti-inflammatory agents aspirin and salicylate inhibit the activity of IκB kinase-β. Nature. 1998;396(6706):77–80. doi:10.1038/23948. 9817203

[33] Dovizio M, Bruno A, Tacconelli S, Patrignani P. Mode of action of aspirin as a chemopreventive agent. Recent Results Cancer Res. 2013;191:39–65. 22893199 10.1007/978-3-642-30331-9_3

[34] Ying J, Zhou H, Liu P, You Q, Kuang F, Shen Y, Hu Z. Aspirin inhibited the metastasis of colon cancer cells by inhibiting the expression of toll-like receptor 4. Cell Biosci. 2018;8(1). doi:10.1186/s13578-017-0198-7. PMC5753438 29308184

[35] Jung YR, Kim EJ, Choi HJ, Park -J-J, Kim H-S, Lee Y-J, Park M-J, Lee M. Aspirin targets SIRT1 and AMPK to induce senescence of colorectal carcinoma cells. Mol Pharmacol. 2015;88(4):708–719. doi:10.1124/mol.115.098616. 26219912

[36] Din FVN, Valanciute A, Houde VP, Zibrova D, Green KA, Sakamoto K, Alessi DR, Dunlop MG. Aspirin inhibits mTOR signaling, activates AMP-activated protein kinase, and induces autophagy in colorectal cancer cells. Gastroenterology. 2012;142(7):1504–15.e3. doi:10.1053/j.gastro.2012.02.050. 22406476 PMC3682211

[37] Zimmermann KC, Waterhouse NJ, Goldstein JC, Schuler M, Green DR. Aspirin induces apoptosis through release of cytochrome c from mitochondria. Neoplasia. 2000;2(6):505–513. doi:10.1038/sj.neo.7900120. 11228543 PMC1508093

[38] Gu Q, Wang JD, Xia HHX, Lin MCM, He H, Zou B, Tu SP, Yang Y, Liu XG, Lam SK, et al. Activation of the caspase-8/Bid and Bax pathways in aspirin-induced apoptosis in gastric cancer. Carcinogenesis. 2005;26(3):541–546. doi:10.1093/carcin/bgh345. 15579484

[39] Wang Y, Du C, Zhang N, Li M, Liu Y, Zhao M, Wang F, Luo F. TGF-β1 mediates the effects of aspirin on colonic tumor cell proliferation and apoptosis. Oncol Lett. 2018;15(4):5903–5909. doi:10.3892/ol.2018.8047. 29552221 PMC5840675

[40] Dovizio M, Maier TJ, Alberti S, Di Francesco L, Marcantoni E, Münch G, John CM, Suess B, Sgambato A, Steinhilber D, et al. Pharmacological inhibition of platelet-tumor cell cross-talk prevents platelet-induced overexpression of cyclooxygenase-2 in HT29 human colon carcinoma cells. Mol Pharmacol. 2013;84(1):25–40. doi:10.1124/mol.113.084988. 23580446 PMC11037430

[41] Patrono C. The multifaceted clinical readouts of platelet inhibition by low-dose aspirin. J Am Coll Cardiol. 2015;66(1):74–85. doi:10.1016/j.jacc.2015.05.012. 26139061

[42] Boutaud O, Sosa IR, Amin T, Oram D, Adler D, Hwang HS, Crews BC, Milne G, Harris BK, Hoeksema M, et al. Inhibition of the biosynthesis of prostaglandin E2 by low-dose aspirin: implications for adenocarcinoma metastasis. Cancer Prev Res (Phila). 2016;9(11):855–865. doi:10.1158/1940-6207.CAPR-16-0094. 27554763 PMC5093073

[43] Mitrugno A, Sylman JL, Ngo ATP, Pang J, Sears RC, Williams CD, McCarty OJT. Aspirin therapy reduces the ability of platelets to promote colon and pancreatic cancer cell proliferation: implications for the oncoprotein c-MYC. Am J Physiol Cell Physiol. 2017;312(2):C176–89. doi:10.1152/ajpcell.00196.2016. 27903583 PMC5336594

[44] Xu XR, Yousef GM, Ni H. Cancer and platelet crosstalk: opportunities and challenges for aspirin and other antiplatelet agents. Blood. 2018;131(16):1777–1789. doi:10.1182/blood-2017-05-743187. 29519806

[45] Kopp H-G, Placke T, Salih HR. Platelet-derived transforming growth factor-β down-regulates NKG2D thereby inhibiting natural killer cell antitumor reactivity. Cancer Res. 2009;69(19):7775–7783. doi:10.1158/0008-5472.CAN-09-2123. 19738039

[46] Placke T, Örgel M, Schaller M, Jung G, Rammensee H-G, Kopp H-G, Salih HR. Platelet-derived MHC class I confers a pseudonormal phenotype to cancer cells that subverts the antitumor reactivity of natural killer immune cells. Cancer Res. 2012;72(2):440–448. doi:10.1158/0008-5472.CAN-11-1872. 22127925

[47] Gay LJ, Felding-Habermann B. Contribution of platelets to tumour metastasis. Nat Rev Cancer. 2011;11(2):123–134. doi:10.1038/nrc3004. 21258396 PMC6894505

[48] Schumacher D, Strilic B, Sivaraj KK, Wettschureck N, Offermanns S. Platelet-derived nucleotides promote tumor-cell transendothelial migration and metastasis via P2Y2 receptor. Cancer Cell. 2013;24(1):130–137. doi:10.1016/j.ccr.2013.05.008. 23810565

[49] Labelle M, Begum S, Hynes RO. Direct signaling between platelets and cancer cells induces an epithelial-mesenchymal-like transition and promotes metastasis. Cancer Cell. 2011;20(5):576–590. doi:10.1016/j.ccr.2011.09.009. 22094253 PMC3487108

[50] Guillem-Llobat P, Dovizio M, Bruno A, Ricciotti E, Cufino V, Sacco A, Grande R, Alberti S, Arena V, Cirillo M, et al. Aspirin prevents colorectal cancer metastasis in mice by splitting the crosstalk between platelets and tumor cells. Oncotarget. 2016;7(22):32462–32477. doi:10.18632/oncotarget.8655. 27074574 PMC5078026

[51] Schottenfeld D, Beebe-Dimmer J. Chronic inflammation: a common and important factor in the pathogenesis of neoplasia. CA Cancer J Clin. 2006;56(2):69–83. doi:10.3322/canjclin.56.2.69. 16514135

[52] Aoki T, Narumiya S. Prostaglandin E2-EP2 signaling as a node of chronic inflammation in the colon tumor microenvironment. Inflammation and Regeneration. 2017;37(1). doi:10.1186/s41232-017-0036-7. PMC5725845 29259703

[53] Ma X, Aoki T, Tsuruyama T, Narumiya S. Definition of prostaglandin E2–EP2 signals in the colon tumor microenvironment that amplify inflammation and tumor growth. Cancer Res. 2015;75(14):2822–2832. doi:10.1158/0008-5472.CAN-15-0125. 26018088

[54] Mizuno R, Kawada K, Sakai Y. Prostaglandin E2/EP signaling in the tumor microenvironment of colorectal cancer. Int J Mol Sci. 2019;20(24):6254. doi:10.3390/ijms20246254. PMC6940958 31835815

[55] Wang D, DuBois RN. The role of COX-2 in intestinal inflammation and colorectal cancer. Oncogene. 2010;29(6):781–788. doi:10.1038/onc.2009.421. 19946329 PMC3181054

[56] Yao C, Sakata D, Esaki Y, Li Y, Matsuoka T, Kuroiwa K, Sugimoto Y, Narumiya S. Prostaglandin E2–EP4 signaling promotes immune inflammation through TH1 cell differentiation and TH17 cell expansion. Nat Med. 2009;15(6):633–640. doi:10.1038/nm.1968. 19465928

[57] Sheibanie AF, Yen J-H, Khayrullina T, Emig F, Zhang M, Tuma R, Ganea D. The proinflammatory effect of prostaglandin E2 in experimental inflammatory bowel disease is mediated through the IL-23→IL-17 axis. J Immunol. 2007;178(12):8138–8147. doi:10.4049/jimmunol.178.12.8138. 17548652

[58] Scandella E, Men Y, Gillessen S, Förster R, Groettrup M. Prostaglandin E2 is a key factor for CCR7 surface expression and migration of monocyte-derived dendritic cells. Blood. 2002;100(4):1354–1361. doi:10.1182/blood-2001-11-0017. 12149218

[59] Stryker SJ, Wolff BG, Culp CE, Libbe SD, Ilstrup DM, MacCarty RL. Natural history of untreated colonic polyps. Gastroenterology. 1987;93(5):1009–1013. doi:10.1016/0016-5085(87)90563-4. 3653628

[60] Sandler RS, Halabi S, Baron JA, Budinger S, Paskett E, Keresztes R, Petrelli N, Pipas JM, Karp DD, Loprinzi CL, et al. A randomized trial of aspirin to prevent colorectal adenomas in patients with previous colorectal cancer. N Engl J Med. 2003;348(10):883–890. doi:10.1056/NEJMoa021633. 12621132

[61] Baron JA, Cole BF, Sandler RS, Haile RW, Ahnen D, Bresalier R, McKeown-Eyssen G, Summers RW, Rothstein R, Burke CA, et al. A randomized trial of aspirin to prevent colorectal adenomas. N Engl J Med. 2003;348(10):891–899. doi:10.1056/NEJMoa021735. 12621133

[62] Logan RFA, Grainge MJ, Shepherd VC, Armitage NC, Muir KR, ukCAP Trial Group. Aspirin and folic acid for the prevention of recurrent colorectal adenomas. Gastroenterology. 2008;134(1):29–38. 10.1053/j.gastro.2007.10.014 18022173

[63] Benamouzig R, Uzzan B, Deyra J, Martin A, Girard B, Little J, Chaussade S, Association pour la Prévention par l’Aspirine du Cancer Colorectal Study Group (APACC). Prevention by daily soluble aspirin of colorectal adenoma recurrence: 4-year results of the APACC randomised trial. Gut. 2012;61(2):255–261. 10.1136/gutjnl-2011-300113 21890814

[64] Ishikawa H, Mutoh M, Suzuki S, Tokudome S, Saida Y, Abe T, Okamura S, Tajika M, Joh T, Tanaka S, et al. The preventive effects of low-dose enteric-coated aspirin tablets on the development of colorectal tumours in Asian patients: a randomised trial. Gut. 2014;63(11):1755–1759. doi:10.1136/gutjnl-2013-305827. 24488498

[65] Pommergaard H-C, Burcharth J, Rosenberg J, Raskov H. Aspirin, calcitriol, and calcium do not prevent adenoma recurrence in a randomized controlled trial. Gastroenterology. 2016;150(1):114–122.e4. doi:10.1053/j.gastro.2015.09.010. 26404953

[66] Hull MA, Sprange K, Hepburn T, Tan W, Shafayat A, Rees CJ, Clifford G, Logan RF, Loadman PM, Williams EA, et al. Eicosapentaenoic acid and aspirin, alone and in combination, for the prevention of colorectal adenomas (seAFOod Polyp Prevention trial): a multicentre, randomised, double-blind, placebo-controlled, 2 × 2 factorial trial. Lancet. 2018;392(10164):2583–2594. doi:10.1016/S0140-6736(18)31775-6. 30466866 PMC6294731

[67] Veettil SK, Lim KG, Ching SM, Saokaew S, Phisalprapa P, Chaiyakunapruk N. Effects of aspirin and non-aspirin nonsteroidal anti-inflammatory drugs on the incidence of recurrent colorectal adenomas: a systematic review with meta-analysis and trial sequential analysis of randomized clinical trials. BMC Cancer. 2017;17(1). doi:10.1186/s12885-017-3757-8. PMC5686945 29137605

[68] Higurashi T, Hosono K, Takahashi H, Komiya Y, Umezawa S, Sakai E, Uchiyama T, Taniguchi L, Hata Y, Uchiyama S, et al. Metformin for chemoprevention of metachronous colorectal adenoma or polyps in post-polypectomy patients without diabetes: a multicentre double-blind, placebo-controlled, randomised phase 3 trial. Lancet Oncol. 2016;17(4):475–483. doi:10.1016/S1470-2045(15)00565-3. 26947328

[69] Friis S, Riis AH, Erichsen R, Baron JA, Sørensen HT. Low-dose aspirin or nonsteroidal anti-inflammatory drug use and colorectal cancer risk: a population-based, case-control study. Ann Intern Med. 2015;163(5):347–355. doi:10.7326/M15-0039. 26302241

[70] Rothwell PM, Wilson M, Elwin C-E, Norrving B, Algra A, Warlow CP, Meade TW. Long-term effect of aspirin on colorectal cancer incidence and mortality: 20-year follow-up of five randomised trials. Lancet. 2010;376(9754):1741–1750. doi:10.1016/S0140-6736(10)61543-7. 20970847

[71] García Rodríguez LA, Soriano-Gabarró M, Bromley S, Lanas A, Cea Soriano L. New use of low-dose aspirin and risk of colorectal cancer by stage at diagnosis: a nested case–control study in UK general practice. BMC Cancer. 2017;17(1). doi:10.1186/s12885-017-3594-9. PMC5590216 28882113

[72] Stürmer T, Glynn RJ, Lee IM, Manson JE, Buring JE, Hennekens CH. Aspirin use and colorectal cancer: post-trial follow-up data from the Physicians’ Health Study. Ann Intern Med. 1998;128(9):713–720. doi:10.7326/0003-4819-128-9-199805010-00003. 9556464

[73] Cook NR, Lee I-M, Gaziano JM, Gordon D, Ridker PM, Manson JE, Hennekens CH, Buring JE. Low-dose aspirin in the primary prevention of cancer: the Women’s Health Study: a randomized controlled trial. JAMA. 2005;294(1):47–55. doi:10.1001/jama.294.1.47. 15998890

[74] Cook NR, Lee I-M, Zhang SM, Moorthy MV, Buring JE. Alternate-day, low-dose aspirin and cancer risk: long-term observational follow-up of a randomized trial. Ann Intern Med. 2013;159(2):77–85. doi:10.7326/0003-4819-159-2-201307160-00002. 23856681 PMC3713531

[75] McNeil JJ, Nelson MR, Woods RL, Lockery JE, Wolfe R, Reid CM, Kirpach B, Shah RC, Ives DG, Storey E, et al. Effect of aspirin on all-cause mortality in the healthy elderly. N Engl J Med. 2018;379(16):1519–1528. doi:10.1056/NEJMoa1803955. 30221595 PMC6433466

[76] Chan AT, Ladabaum U. Where do we stand with aspirin for the prevention of colorectal cancer? The USPSTF recommendations. Gastroenterology. 2016;150(1):14–18. doi:10.1053/j.gastro.2015.11.018. 26602220

[77] Burn J, Bishop DT, Chapman PD, Elliott F, Bertario L, Dunlop MG, Eccles D, Ellis A, Evans DG, Fodde R, et al. A randomized placebo-controlled prevention trial of aspirin and/or resistant starch in young people with familial adenomatous polyposis. Cancer Prev Res (Phila). 2011;4(5):655–665. doi:10.1158/1940-6207.CAPR-11-0106. 21543343 PMC3092423

[78] Burn J, Gerdes A-M, Macrae F, Mecklin J-P, Moeslein G, Olschwang S, Eccles D, Evans DG, Maher ER, Bertario L, et al. Long-term effect of aspirin on cancer risk in carriers of hereditary colorectal cancer: an analysis from the CAPP2 randomised controlled trial. Lancet. 2011;378(9809):2081–2087. doi:10.1016/S0140-6736(11)61049-0. 22036019 PMC3243929

[79] Burn J, Sheth H, Elliott F, Reed L, Macrae F, Mecklin J-P, Möslein G, McRonald FE, Bertario L, Evans DG, et al. Cancer prevention with aspirin in hereditary colorectal cancer (Lynch syndrome), 10-year follow-up and registry-based 20-year data in the CAPP2 study: a double-blind, randomised, placebo-controlled trial. Lancet. 2020;395(10240):1855–1863. doi:10.1016/S0140-6736(20)30366-4. 32534647 PMC7294238

[80] Burn J, Mathers JC, Bishop DT. Chemoprevention in Lynch syndrome. Fam Cancer. 2013;12(4):707–718. doi:10.1007/s10689-013-9650-y. 23880960

[81] Effect of chemoprevention by low-dose aspirin of new or recurrent colorectal adenomas in patients with lynch syndrome - full text view - ClinicalTrials.gov; 2020. [accedded 2020 Apr 30 https://clinicaltrials.gov/ct2/show/NCT02813824.

[82] Chan AT. Aspirin use and survival after diagnosis of colorectal cancer. JAMA. 2009;302(6):649–658. doi:10.1001/jama.2009.1112. 19671906 PMC2848289

[83] Restivo A, Cocco IMF, Casula G, Scintu F, Cabras F, Scartozzi M, Zorcolo L. Aspirin as a neoadjuvant agent during preoperative chemoradiation for rectal cancer. Br J Cancer. 2015;113(8):1133–1139. doi:10.1038/bjc.2015.336. 26372700 PMC4647877

[84] Rothwell PM, Wilson M, Price JF, Belch JF, Meade TW, Mehta Z. Effect of daily aspirin on risk of cancer metastasis: a study of incident cancers during randomised controlled trials. The Lancet. 2012;379(9826):1591–1601. doi:10.1016/S0140-6736(12)60209-8. 22440947

[85] Yang L, Lv Z, Xia W, Zhang W, Xin Y, Yuan H, Chen Y, Hu X, Lv Y, Xu Q, et al. The effect of aspirin on circulating tumor cells in metastatic colorectal and breast cancer patients: a phase II trial study. Clin Transl Oncol. 2018;20(7):912–921. doi:10.1007/s12094-017-1806-z. 29243075

[86] Li P, Wu H, Zhang H, Shi Y, Xu J, Ye Y, Xia D, Yang J, Cai J, Wu Y. Aspirin use after diagnosis but not prediagnosis improves established colorectal cancer survival: a meta-analysis. Gut. 2015;64(9):1419–1425. doi:10.1136/gutjnl-2014-308260. 25239119

[87] Bastiaannet E, Sampieri K, Dekkers OM, de Craen AJM, van Herk-sukel MPP, Lemmens V, van den Broek CBM, Coebergh JW, Herings RMC, van de Velde CJH, et al. Use of aspirin postdiagnosis improves survival for colon cancer patients. Br J Cancer. 2012;106(9):1564–1570. doi:10.1038/bjc.2012.101. 22454078 PMC3341868

[88] McCowan C, Munro AJ, Donnan PT, Steele RJC. Use of aspirin post-diagnosis in a cohort of patients with colorectal cancer and its association with all-cause and colorectal cancer specific mortality. Eur J Cancer. 2013;49(5):1049–1057. doi:10.1016/j.ejca.2012.10.024. 23182687

[89] Goh CH, Goh HH, Leong WQ, Chew MH, Pan YS, Tony LKH, Chew L, Tan IBH, Toh HC, Tang CL, et al. Post-operative aspirin use and colorectal cancer-specific survival in patients with stage I-III colorectal cancer. Anticancer Res. 2014;34(12):7407–7414. 25503181

[90] Bains SJ, Mahic M, Myklebust TÅ, Småstuen MC, Yaqub S, Dørum LM, Bjørnbeth BA, Møller B, Brudvik KW, Taskén K. Aspirin as secondary prevention in patients with colorectal cancer: an unselected population-based study. J Clin Oncol. 2016;34(21):2501–2508. doi:10.1200/JCO.2015.65.3519. 27247217

[91] Cardwell CR, Kunzmann AT, Cantwell MM, Hughes C, Baron JA, Powe DG, Murray LJ. Low-dose aspirin use after diagnosis of colorectal cancer does not increase survival: a case–control analysis of a population-based cohort. Gastroenterology. 2014;146(3):700–708.e2. doi:10.1053/j.gastro.2013.11.005. 24239563

[92] Gray RT, Coleman HG, Hughes C, Murray LJ, Cardwell CR. Low-dose aspirin use and survival in colorectal cancer: results from a population-based cohort study. BMC Cancer. 2018;18(1):228. doi:10.1186/s12885-018-4142-y. 29486728 PMC6389196

[93] Ali R, Toh H-C, Chia W-K. The utility of Aspirin in dukes C and high risk dukes B colorectal cancer - The ASCOLT study: study protocol for a randomized controlled trial. Trials. 2011;12(1):261. doi:10.1186/1745-6215-12-261. 22168568 PMC3271983

[94] Michel P, Boige V, Andre T, Aparicio T, Bachet JB, Dahan L, Guimbaud R, Lepage C, Manfredi S, Tougeron D, et al. Aspirin versus placebo in stage III or high-risk stage II colon cancer with PIK3CA mutation: a French randomised double-blind phase III trial (PRODIGE 50-ASPIK). Dig Liver Dis. 2018;50(3):305–307. doi:10.1016/j.dld.2017.12.023. 29402752

[95] Aspirin for prevention of postsurgical recurrence and metastasis in asian colorectal cancer patients: a multi-center randomized trial - full text view - ClinicalTrials.gov; 2020. [cited 2020 Apr 30]. https://clinicaltrials.gov/ct2/show/NCT02607072.

[96] Coyle C, Cafferty FH, Rowley S, MacKenzie M, Berkman L, Gupta S, Pramesh CS, Gilbert D, Kynaston H, Cameron D, et al. ADD-ASPIRIN: a phase III, double-blind, placebo controlled, randomised trial assessing the effects of aspirin on disease recurrence and survival after primary therapy in common non-metastatic solid tumours. Contemp Clin Trials. 2016;51:56–64. doi:10.1016/j.cct.2016.10.004. 27777129 PMC5127874

[97] Nan H, Hutter CM, Lin Y, Jacobs EJ, Ulrich CM, White E, Baron JA, Berndt SI, Brenner H, Butterbach K, et al. Association of Aspirin and NSAID use with risk of colorectal cancer according to genetic variants. JAMA. 2015;313(11):1133–1142. doi:10.1001/jama.2015.1815. 25781442 PMC4382867

[98] Nan H, Morikawa T, Suuriniemi M, Imamura Y, Werner L, Kuchiba A, Yamauchi M, Hunter DJ, Kraft P, Giovannucci EL, et al. Aspirin use, 8q24 single nucleotide polymorphism rs6983267, and colorectal cancer according to CTNNB1 alterations. J Natl Cancer Inst. 2013;105(24):1852–1861. doi:10.1093/jnci/djt331. 24317174 PMC3866156

[99] Resler AJ, Makar KW, Heath L, Whitton J, Potter JD, Poole EM, Habermann N, Scherer D, Duggan D, Wang H, et al. Genetic variation in prostaglandin synthesis and related pathways, NSAID use and colorectal cancer risk in the Colon Cancer Family Registry. Carcinogenesis. 2014;35(9):2121–2126. doi:10.1093/carcin/bgu119. 24908683 PMC4146420

[100] Hubner RA, Muir KR, Liu J-F, Logan RFA, Grainge MJ, Houlston RS. Members of the UKCAP Consortium. Ornithine decarboxylase G316A genotype is prognostic for colorectal adenoma recurrence and predicts efficacy of aspirin chemoprevention. Clin Cancer Res. 2008;14(8):2303–2309. doi:10.1158/1078-0432.CCR-07-4599. 18413818

[101] Barry EL, Mott LA, Sandler RS, Ahnen DJ, Baron JA. Variants downstream of the ornithine decarboxylase gene influence risk of colorectal adenoma and Aspirin Chemoprevention. Cancer Prev Res (Phila). 2011;4(12):2072–2082. doi:10.1158/1940-6207.CAPR-11-0300. 21930798 PMC3232321

[102] Gray RT, Cantwell MM, Coleman HG, Loughrey MB, Bankhead P, McQuaid S, O’Neill RF, Arthur K, Bingham V, McGready C, et al. Evaluation of PTGS2 expression, PIK3CA mutation, aspirin use and colon cancer survival in a population-based cohort study. Clin Transl Gastroenterol. 2017;8(4):e91. doi:10.1038/ctg.2017.18. 28448072 PMC5543466

[103] Shrubsole MJ, Cai Q, Wen W, Milne G, Smalley WE, Chen Z, Ness RM, Zheng W. Urinary prostaglandin E2 metabolite and risk for colorectal adenoma. Cancer Prev Res (Phila). 2012;5(2):336–342. doi:10.1158/1940-6207.CAPR-11-0426. 22166248 PMC3273609

[104] Bezawada N, Song M, Wu K, Mehta RS, Milne GL, Ogino S, Fuchs CS, Giovannucci EL, Chan AT. Urinary PGE-M levels are associated with risk of colorectal adenomas and chemopreventive response to anti-inflammatory drugs. Cancer Prev Res (Phila). 2014;7(7):758–765. doi:10.1158/1940-6207.CAPR-14-0120. 24824037 PMC4085181

[105] Fink SP, Yamauchi M, Nishihara R, Jung S, Kuchiba A, Wu K, Cho E, Giovannucci E, Fuchs CS, Ogino S, et al. Aspirin and the risk of colorectal cancer in relation to the expression of 15-hydroxyprostaglandin dehydrogenase (HPGD). Sci Transl Med. 2014;6(233):233re2. doi:10.1126/scitranslmed.3008481. PMC4030641 24760190

[106] Fink SP, Yang D-H, Barnholtz-Sloan JS, Ryu Y-M, Mikkola D, Potter JD, Lampe JW, Markowitz SD, Myung S-J. Colonic 15-PGDH levels are stable across distance and time and are not perturbed by aspirin intervention. Dig Dis Sci. 2013;58(9):2615–2622. doi:10.1007/s10620-013-2670-5. 23625286 PMC3769508

[107] Nishihara R, Lochhead P, Kuchiba A, Jung S, Yamauchi M, Liao X, Imamura Y, Qian ZR, Morikawa T, Wang M, et al. Aspirin use and risk of colorectal cancer according to braf mutation status. JAMA. 2013;309(24):2563–2571. doi:10.1001/jama.2013.6599. 23800934 PMC3743040

[108] Hua X, Phipps AI, Burnett-Hartman AN, Adams SV, Hardikar S, Cohen SA, Kocarnik JM, Ahnen DJ, Lindor NM, Baron JA, et al. Timing of aspirin and other nonsteroidal anti-inflammatory drug use among patients with colorectal cancer in relation to tumor markers and survival. J Clin Oncol. 2017;35(24):2806–2813. doi:10.1200/JCO.2017.72.3569. 28617623 PMC5562174

[109] Liao X, Lochhead P, Nishihara R, Morikawa T, Kuchiba A, Yamauchi M, Imamura Y, Qian ZR, Baba Y, Shima K, et al. Aspirin use, tumor PIK3CA mutation, and colorectal-cancer survival. N Engl J Med. 2012;367(17):1596–1606. doi:10.1056/NEJMoa1207756. 23094721 PMC3532946

[110] Domingo E, Church DN, Sieber O, Ramamoorthy R, Yanagisawa Y, Johnstone E, Davidson B, Kerr DJ, Tomlinson IPM, Midgley R. Evaluation of PIK3CA mutation as a predictor of benefit from nonsteroidal anti-inflammatory drug therapy in colorectal cancer. J Clin Oncol. 2013;31(34):4297–4305. doi:10.1200/JCO.2013.50.0322. 24062397

[111] Reimers MS, Bastiaannet E, Langley RE, van Eijk R, van Vlierberghe RLP, Lemmens VEP, van Herk-sukel MPP, van Wezel T, Fodde R, Kuppen PJK, et al. Expression of HLA class I antigen, aspirin use, and survival after a diagnosis of colon cancer. JAMA Intern Med. 2014;174(5):732–739. doi:10.1001/jamainternmed.2014.511. 24687028

[112] Kothari N, Kim R, Jorissen RN, Desai J, Tie J, Wong H-L, Farragher I, Jones I, Day FL, LI S, et al. Impact of regular aspirin use on overall and cancer-specific survival in patients with colorectal cancer harboring a PIK3CA mutation. Acta Oncol. 2015;54(4):487–492. doi:10.3109/0284186X.2014.990158. 25549537 PMC4743650

[113] Murphy C, Turner N, Wong H-L, Sinnathamby M, Tie J, Lee B, Desai J, Skinner I, Christie M, Hutchinson R, et al. Examining the impact of regular aspirin use and PIK3CA mutations on survival in stage 2 colon cancer. Intern Med J. 2017;47(1):88–98. doi:10.1111/imj.13312. 27800646

[114] Faivre J, Dancourt V, Lejeune C, Tazi MA, Lamour J, Gerard D, Dassonville F, Bonithon-Kopp C. Reduction in colorectal cancer mortality by fecal occult blood screening in a French controlled study. Gastroenterology. 2004;126(7):1674–1680. doi:10.1053/j.gastro.2004.02.018. 15188160

[115] Brenner H, Calderazzo S, Seufferlein T, Ludwig L, Dikopoulos N, Mangold J, Böck W, Stolz T, Eisenbach T, Block T, et al. Effect of a single aspirin dose prior to fecal immunochemical testing on test sensitivity for detecting advanced colorectal neoplasms. JAMA. 2019;321(17):1686–1692. doi:10.1001/jama.2019.4755. 31063574 PMC6506873

[116] Randel KR, Botteri E, Romstad KMK, Frigstad SO, Bretthauer M, Hoff G, de Lange T, Holme Ø. Effects of oral anticoagulants and aspirin on performance of fecal immunochemical tests in colorectal cancer screening. Gastroenterology. 2019;156(6):1642–1649.e1. doi:10.1053/j.gastro.2019.01.040. 30689972

[117] Nieuwenburg SAV, Vuik FER, Kruip MJHA, Kuipers EJ, Spaander MCW. Effect of anticoagulants and NSAIDs on accuracy of faecal immunochemical tests (FITs) in colorectal cancer screening: a systematic review and meta-analysis. Gut. 2019;68(5):866–872. doi:10.1136/gutjnl-2018-316344. 29871970

[118] Robertson DJ, Lee JK, Boland CR, Dominitz JA, Giardiello FM, Johnson DA, Kaltenbach T, Lieberman D, Levin TR, Rex DK. Recommendations on fecal immunochemical testing to screen for colorectal neoplasia: a consensus statement by the US multi-society task force on colorectal cancer. Gastroenterology. 2017;152(5):1217–1237.e3. doi:10.1053/j.gastro.2016.08.053. 27769517

[119] Tsoi KK, Chan FC, Hirai HW, Sung JJ. Risk of gastrointestinal bleeding and benefit from colorectal cancer reduction from long-term use of low-dose aspirin: a retrospective study of 612 509 patients. J Gastroenterol Hepatol. 2018;33(10):1728–1736. doi:10.1111/jgh.14261. 29665624

[120] Lanas A, Wu P, Medin J, Mills EJ. Low doses of acetylsalicylic acid increase risk of gastrointestinal bleeding in a meta-analysis. Clinical Gastroenterology and Hepatology. 2011;9(9):762–768.e6. doi:10.1016/j.cgh.2011.05.020. 21699808

[121] Serebruany VL, Steinhubl SR, Berger PB, Malinin AI, Baggish JS, Bhatt DL, Topol EJ. Analysis of risk of bleeding complications after different doses of aspirin in 192,036 patients enrolled in 31 randomized controlled trials. Am J Cardiol. 2005;95(10):1218–1222. doi:10.1016/j.amjcard.2005.01.049. 15877994

