# Supplementary material for: Colorectal cancer chemoprevention: is aspirin still in the game?
Source: Cancer Biol Ther. 2022 Jul 29;23(1):446–61. doi: 10.1080/15384047.2022.2104561 (PMC9341367; doi:10.1080/15384047.2022.2104561)
Supplement: Supplemental Material [file KCBT_A_2104561_SM3705.pdf]

Taylor & Francis
Taylor & Francis Group

Journal Publication Costs Form

Taylor & Francis, 530 Walnut Street, Suite 850 Philadelphia, PA 19106

Date: July 15, 2022

Journal title: Cancer Biology & Therapy

Manuscript Identification Number: KCBT-2021-028 **Article Type:** review

Manuscript Title: Colorectal cancer chemoprevention : is aspirin still in the game ?

Corresponding Author Name: Adrien Grancher

Corresponding Author Email: grancher.adr@gmail.com

Please consult the journal's Instructions for Authors page via https://www.tandfonline.com/ to confirm the cost of printing images in color. Failure to return a completed form will result in delayed publication if your article is accepted. Please note that the formal invoice for charges applicable to your paper will be raised by Production if your paper is accepted. If your manuscript is not accepted, you will not be charged.

Color Figures

Optional cost for print issues only. By checking this box you confirm that color figures should be published in color and color charges should be added to your invoice: ✔

If choosing to print in color, please list which images should be in color and include the Figure number and caption:

Figure 1

Billing Address Information

Please provide complete and legible billing address information below. The person listed here will receive the invoice for all applicable charges (page charges, color charges, or Open Access charges).

Name: Dr Adrien Grancher

Institution: Department of Hepatogastroenterology Rouen University H

Address: 1 rue de Germont

City/State/Post or Zip code: 76000 ROUEN

Country: France

Phone: 06 73 08 36 11

Email (required): grancher.adr@gmail.com

Purchase Order Number (if applicable): ______

Return completed form to the Journal Editorial Office

Information Classification: General
